# 29-year-old Woman with Dyspnea

**DOI:** 10.5811/cpcem.2017.6.34490

**Published:** 2017-07-17

**Authors:** Elizabeth England, Michele Callahan, Laura J. Bontempo, Zachary D.W. Dezman

**Affiliations:** * University of Maryland Medical Center, Baltimore, Maryland; † University of Maryland School of Medicine, Department of Emergency Medicine, Baltimore, Maryland

## CASE PRESENTATION

A 29-year-old female presented to the emergency department (ED) with a chief complaint of worsening dyspnea over the prior three weeks. Her shortness of breath was exacerbated by exertion and lying down. It was also worse at night. Over the same time, she had developed a dry, raspy, non-productive cough, bilateral leg swelling, and chest tightness. She denied any fevers, chest or abdominal pain, recent travel, or viral illness. She had no medical problems or past surgical history. Her only home medication was ibuprofen and she had no known drug allergies. She denied any family history of sudden death, myocardial infarction, or heart failure. She denied tobacco or illicit drug use. She reported occasionally drinking alcohol. She had been employed as a welder for the past three years and had recently increased her work hours.

The patient had an initial blood pressure of 138/108 mmHg, heart rate of 126 beats per minute, respiratory rate of 18 breaths per minute, temperature of 36.7° Celsius, and an oxygen saturation of 99% on room air. Shortly after being placed in a room, the patient desaturated to 88% on room air. She was placed on two liters per minute of oxygen by nasal cannula, with improvement of her saturation to 95%. On physical exam, the patient was well developed, well nourished, and appeared to be her stated age. She was in no acute distress. Her head, eye, ear, nose and throat exams were all unremarkable. Neck exam showed no jugular vein distention and no goiter. On cardiac exam, she was found to be tachycardic with a regular rhythm and an audible s3 gallop. She was tachypneic without accessory muscle use. Rales were heard in all lung fields. Abdominal exam was unremarkable. She was noted to have trace pedal edema with normal range of motion of her joints and limbs. She was awake, alert and appropriately interactive without focality to her neurological examination. Skin examination showed no rashes or erythema.

**Image 1:**
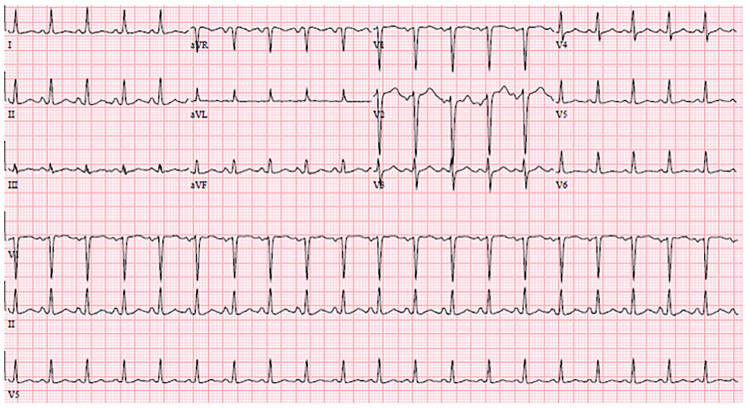
Electrocardiogram on arrival to the emergency department.

**Image 2:**
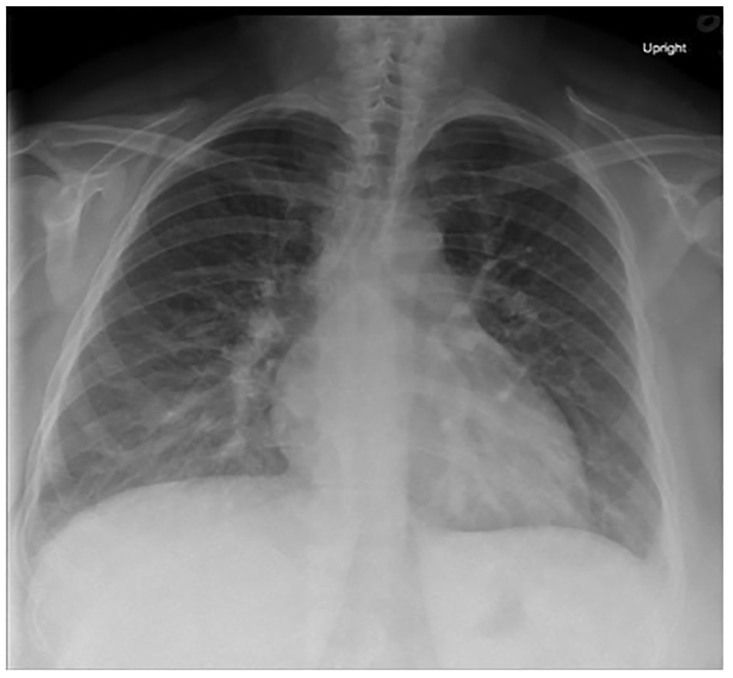
Initial anterior-posterior chest radiograph taken shortly after arrival to the emergency department.

Her laboratory results are shown in Tables 1–3. Her electrocardiogram (ECG) and chest radiography are shown in Images 1 and 2. A bedside ultrasound (US) was performed and was notable for B-lines bilaterally and a grossly decreased ejection fraction, estimated to be approximately 20%. Following initial physician evaluation, the patient desaturated to 92% on nasal cannula and her oxygen flow was increased to four liters per minute. Shortly thereafter she was started on bilevel positive airway pressure (BiPAP) and given nitroglycerin, enalapril, and furosemide. This resulted in significant improvement in her symptoms. She was taken off BiPAP and had an oxygen saturation of 96% on three liters nasal cannula. She was admitted to the intermediate care unit. A diagnostic test was then performed, which confirmed her diagnosis.

## DISCUSSION

When we were first presented with this patient’s case, several key points within the history of present illness jumped out immediately. First of all, this was a 29-year-old female presenting with fairly insidious onset of shortness of breath. Her report of dyspnea on exertion, bilateral lower extremity swelling, non-productive cough, and orthopnea seemed more fitting for a middle-aged patient than someone who had not yet reached 30 years of age.

A few additional pieces of information struck me as important. Her lack of a family history of early coronary artery disease or sudden death, although helpful in risk-stratifying many patients who present with chest pain or dyspnea, did not help to clarify the potential cause for her symptoms. When asked about social history, she reported only occasional alcohol use. This led me to question – was she being truthful? Could this be a disease process in the setting of heavier, longer-term alcohol abuse? I would certainly probe her for more specific details about her alcohol intake. In addition, her employment as a welder seemed as if it could play a significant role in her disease process. Different environmental exposures can manifest as pulmonary and cardiac disease, but I am unsure how long she would need to be exposed before these exposures became symptomatic. Including this environmental exposure certainly opens up interesting and rare diagnostic possibilities. Finally, her lack of risk factors, fever, recent travel, and viral prodrome is helpful in placing certain diagnoses, particularly infectious, lower on the differential.

Her physical examination was notable for a narrow pulse pressure, tachycardia, decreased oxygen saturation that responded to oxygen supplementation, tachypnea with rales noted on lung auscultation, a gallop on cardiac auscultation, and pedal edema. Pertinent negatives included being afebrile as well as the lack of a murmur on examination. The rest of her examination was noncontributory.

When approaching the undifferentiated patient with dyspnea, it is helpful to think broadly about all categories of disease processes and then work backwards to see which fit best with the patient’s presentation. There is often a “gut” feeling about what the diagnosis could be – for me, when I first read this case, I immediately felt this was a diagnosis of heart failure. However, approaching patients this way can lead to premature closure, anchoring, and ultimately misdiagnosis. We need to take a step back and explore the differential to ensure we do not miss a critical diagnosis.

Ideally, there should be one final diagnosis that incorporates all of the “positive” and “negative” symptoms that are present or absent, respectively. Using a systematic approach can allow you to explore all of the possibilities and help to avoid missing an important diagnosis (or make an incorrect one). For a patient presenting with dyspnea, there are four broad categories of disease processes: pulmonary, cardiac, neuromuscular, and metabolic.^1^ When exploring these options, we need to ensure that we are including, and evaluating for, the life-threatening causes of dyspnea within our differential.

Let’s begin with discussion of the cardiac causes of dyspnea. Highest on my differential was a form of acute heart failure, possibly from a cardiomyopathy given such a young patient. We will return to this in a moment. Could this be an acute coronary syndrome? This seems less likely in the setting of three weeks of constant symptoms, a non-ischemic ECG, and a troponin level of <0.02 ng/ml. A pulmonary embolism (PE) could account for the dyspnea lasting several weeks (and potentially for right heart failure in the setting of elevated pulmonary pressures), but her risk factors for development of a PE are unclear. She does exhibit sinus tachycardia on the ECG, and thus we cannot apply the pulmonary embolism rule-out criteria (PERC) to this patient. Her ECG does not show any of the right-heart strain patterns that are suggestive of a PE: right bundle-branch block, T-wave inversions in the inferior and anterior leads, right axis deviation, or the classic S1Q3T3 pattern.^2^ Note that these patterns are all specific, and not sensitive. But based on the risk factors presented and my clinical suspicion, I believe she is low risk for a pulmonary embolism. For now we will keep PE as an unlikely diagnosis that should be ruled out.

Valvular diseases may present with dyspnea. This patient does not have a murmur, and no risk factors for endocarditis are included in the history. A bedside US was done, which did not report any vegetations or valvular insufficiency, making valvular disease less likely. Similarly, there was no mention of a pericardial effusion or tamponade. I strongly suspect that if these things were present, they would have been reported. We can therefore also rule out tamponade. The patient’s tachycardia supports myocarditis and/or pericarditis as possible causes of her dyspnea, but she is not pregnant, she did not have the viral prodrome, chest pain, friction rub, or an elevated troponin, despite three weeks of constant symptoms. Her ECG also does not demonstrate the “classic” findings one would expect with pericarditis, such as diffuse PR segment depression that is often most notable in the inferior leads, widespread ST segment elevation, or TP segment downsloping.^2^ As with PE, ECG findings are not present in all cases of pericarditis or myocarditis, but we will move these diagnoses farther down on the list of possibilities. Finally, we know that the patient’s symptoms are not due to a current arrhythmia like atrial fibrillation or atrial flutter based on her ECG, so we will take this off the differential for now.

The patient could have a primary pulmonary cause of her dyspnea such as undiagnosed chronic obstructive pulmonary disease or asthma. But given the patient was asymptomatic until three weeks ago, she does not smoke, there was no wheezing on exam, and her chest radiography does not show any hyperinflation, these diagnoses are less likely. The chest radiograph shows no evidence of a focal infiltrate, making a significant pneumonia unlikely. There is no evidence of a pneumothorax (tension or otherwise) on her chest radiograph or exam. Although the radiograph does demonstrate increased vascular congestion, there are no large pleural effusions noted. The patient demonstrates no signs of an upper airway obstruction such as stridor, drooling, or oropharyngeal edema. It is possible that she has developed a pneumonitis of an unclear cause, but the chest radiography provided shows no evidence of diffuse lung damage or fibrosis. Finally, the possibility of an interstitial lung disease with or without pulmonary hypertension is plausible given her occupational exposure to various materials like arsenic, lead, and mercury, but these tests were all negative and the chest radiograph shows no evidence of diffuse lung fibrosis.

Myasthenia gravis and Guillain-Barre syndrome are two neuromuscular diseases that can present as dyspnea in a young woman, but they are often accompanied by symptoms of generalized muscular weakness. The presence of severe kyphoscoliosis can make it difficult for patients to breathe efficiently, but her exam and chest radiograph show no evidence of significant spinal malformations. She does not manifest any symptoms of bulbar weakness, abnormal reflexes, or difficulty swallowing, and the history of three weeks of symptoms makes diseases such as botulism and poliomyelitis very unlikely. Finally, a stroke may present with difficulty controlling breathing (secondary to muscle weakness), but the patient manifests no other signs or symptoms consistent with this. Therefore, we can remove the neuromuscular and neurologic causes for dyspnea from our list of possibilities.

Finally, let us review potential metabolic and toxicologic causes for this patient’s dyspnea. Anemia and thyrotoxicosis can cause dyspnea, but the laboratory values do not support these diagnoses. Metabolic acidosis should always be considered in patients who are tachypneic or appear dyspneic, as this can be a compensatory mechanism for a primary metabolic acidosis (e.g., diabetic ketoacidosis), but the laboratory results show she is not suffering from one. Anxiety is a fairly common cause for dyspnea, but should be considered a diagnosis of exclusion in the ED. The patient does not appear anxious, and anxiety would not account for the patient’s symptoms of orthopnea, increased lower extremity edema, and poor oxygen saturation. Finally, iatrogenic or pharmacologic causes for dyspnea would include salicylates or beta-blockers, but as the patient denies drug use and only uses ibuprofen as needed, it is unlikely that her dyspnea is related to medication or drug use.

Narrowing down our broad differential, the diagnoses that remain include the following: heart failure (possibly in the setting of a cardiomyopathy), pulmonary embolism, myocarditis +/− pericarditis, and interstitial lung disease in the setting of an occupational exposure. We are given the results of her chest radiograph: increased interstitial markings, prominent hilar vasculature indicative of vascular congestion, interstitial edema, and developing alveolar edema, which is similar to the classic chest radiograph findings of heart failure. We are also given the results of a bedside echocardiogram, which describes an ejection fraction of approximately 20%, a dilated inferior vena cava, and no evidence of pericardial effusion. There is no comment about right heart dilation or other signs that would be concerning for a PE. From the information provided, we can make the following determinations: she has evidence of systolic failure, elevated central venous and right heart pressures, and does not have an obvious obstructive lesion such as tamponade or evidence of a massive PE.

Putting it all together, her historical features of orthopnea and bilateral lower extremity edema have positive likelihood ratios (+LR) of 2.2 and 2.3, respectively, to predict heart failure in patients presenting to the ED with dyspnea.^3^ Her physical exam (S3 gallop +LR=11.0, rales +LR=2.8, edema +LR=2.1) and imaging (chest radiograph with pulmonary venous congestion +LR=12.0) were consistent with a diagnosis of heart failure as well.^3^ We multiply LRs to obtain our post-test probability, but a LR>10 is generally considered diagnostic in common conditions. Ultimately, I believe this patient’s symptoms and presentation are consistent with an episode of newly diagnosed heart failure. The etiology behind the heart failure is unclear. To further elucidate the cause for her symptoms, my recommendation would include both cardiac magnetic resonance imaging (MRI) as well as a myocardial biopsy.

## OUTCOME

Cardiac MRI revealed that the patient had non-ischemic dilated cardiomyopathy. Multiple segments within the mid cavity and apex demonstrated prominent trabeculation. The ratio of non-compacted to compacted muscle was greater than 2.3, suggestive of left ventricular non-compaction cardiomyopathy. The patient was admitted and treated with furosemide, lisinopril, and metoprolol. This resulted in significant improvement. The patient was discharged after a three-day admission with plans for a six-week follow-up echocardiogram.

At follow-up, her echocardiogram demonstrated an ejection fraction of 45–50%. The severity of her symptoms while on therapy are New York Heart Association class IC (Table 4).^4^ She is currently planning on having a child and is working with a genetic counselor to estimate the risk of passing on her condition.

## RESIDENT DISCUSSION

Left ventricular non-compaction cardiomyopathy (LVNCCM) is a rare malformation of embryologic cardiac tissue. While the exact incidence is unknown, 0.014%–1.3% of patients undergoing echocardiography are found to have LVNCCM.^5^ An estimated 3–4% of patients with heart failure have a diagnosis of LVNCCM. With improvement in diagnostic testing and increased awareness and recognition of the disease, it is likely that this incidence will increase.^5^

The pathophysiologic cause of ventricular non-compaction is an area of continued research. The most widely accepted theory is a failure in the process of compaction, proliferation, and organization of the tissue into the adult myocardial architecture, leading to decreased cardiac contractility and function. In the maturing embryo, cardiac tissue goes through several stages of development. Initially, the myocardium consists of two layers of cells that comprise the primitive tubular heart.^5^ Within the first weeks of development, these layers form myocardial protrusions, or trabeculations, which extend into both ventricular cavities. This trabecular formation allows for adequate oxygenation of cardiac tissue prior to development of the coronary arteries by increasing the tissue surface area contact with blood.^5^ As the coronary arteries develop from the epicardium, the trabecular tissue begins to compact and proliferate. This compaction is more prominent in the left ventricle than the right, and right ventricular trabeculations can be seen in the healthy adult.

LVNCCM is diagnosed in various stages of life. A meta-analysis by Bhatia et al. examined 241 adult patients diagnosed with LVNCCM and found it was more prevalent in men than women (65% male), with a mean age of 41 years at the time of diagnosis.^6^ Patients most often presented with symptoms of acute heart failure: shortness of breath (60%), palpitations (18%), and chest pain (15%).^6^ In the pediatric population, patients are more frequently identified during regular screening exams. In a single center study performed by Brescia et al., 240 pediatric patients (median age=9.4 years, interquartile range of 3 months–13.8 years) were diagnosed with LVNCCM.^7^ The most common presentation in symptomatic children was also acute heart failure (25%). However, nearly half of cases were identified through an abnormal screening exam: on physical exam (19%), ECG/chest radiography (16%), or echocardiography (14%).^7^ In both age ranges, significant proportions of patients presented with arrhythmia, syncope, chest pain, and sudden cardiac death.^6^,^7^ In adults, 14% of patients were deceased by 39-month follow-up, half of which were due to sudden cardiac death.^6^ In children, 12.8% of patients had died four years later.^7^

The original and most commonly used diagnostic modality for ventricular non-compaction is echocardiography.^8^ The Jenni criteria remain the most widely accepted criteria for echocardiographic diagnosis. The criteria include bilayered myocardium (a grossly trabeculated or non-compacted layer with a layer of normal, compact myocardium), a non-compacted to compacted ratio >2:1, communications within the intertrabecular space demonstrated by Doppler, absence of coexisting cardiac abnormalities, and presence of multiple prominent trabeculations in end-systole.^8^ Cardiac MRI has become an increasingly popular method to diagnose LVNCCM. A study by Peterson et al. compared healthy volunteers to those diagnosed with LVNCCM.^9^ A ratio of non-compacted:compacted myocardium greater than 2.3 was found to be 86% sensitivity and 99% specificity for LVNCCM.^10^

While non-compaction cardiomyopathy is unlikely to be formally diagnosed in the ED, this case demonstrates how bedside ultrasonography can play a vital role in the evaluation of undifferentiated dyspnea and diagnosis of heart failure. Finding “B-lines” or “comet-tails” on bedside US is 92.5% sensitive and 65.1% specific for interstitial pulmonary edema. Additionally, left ventricular systolic function and volume status (via evaluation of the inferior vena cava) can be estimated for further differentiation of a cardiac or pulmonary cause and help direct medical therapy.^11^

Current recommendations for the initial management of LVNCCM are identical to traditional systolic heart failure management, with a focus on reducing afterload, optimizing volume status, and supporting respiratory function.^12^ For comparison, diastolic heart failure arises from a too-stiff ventricular wall, which does not relax fully in diastole. This leads to decreased filling and decreased cardiac output, leading to dyspnea and other symptoms of heart failure. Long-term management of LVNCCM is largely similar, with continued debate regarding additional need for anticoagulation. At this time, data are largely lacking on the risk for cardioembolic events in LVNCCM as compared with other causes of heart failure. Some experts recommend initiating anticoagulation for patients with either atrial fibrillation or an ejection fraction less than 40%.^12^ A number of cardiologists recommend using a Holter monitor to surveil for arrhythmias or placing an internal cardiac defibrillator because of the high risk of sudden cardiac death in these patients.^7^ As EPs, we play a critical role in improving outcomes in these patients by recognizing and stabilizing their acute heart failure when it occurs.

## FINAL DIAGNOSIS

Final diagnosis was acute heart failure as a result of left ventricular non-compaction cardiomyopathy.

## TAKE-HOME POINTS

- When evaluating an undifferentiated patient with dyspnea, keep a broad differential (i.e., consider more than just cardiac and pulmonary causes) to avoid premature closure or other diagnostic errors.
- Bedside echocardiography is an important skill set for EPs. It allows for the rapid evaluation for many causes of acute dyspnea, including acute heart failure with pulmonary edema, pericardial effusion, or pneumothorax.

## Figures and Tables

**Table 1:** Hematology and coagulation studies of a 29-year-old woman with dyspnea.

Complete blood cell count	
White blood cell count	7.9 K/mcL
Hemoglobin	14.3 g/dL
Hematocrit	41.30%
Platelets	297 K/mcL
Coagulation studies	
International normalized ratio	1.0
Prothrombin time	13.1 sec
Activated partial thromboplastin time	28 sec

**Table 2:** Chemistry results.

Sodium	142 mmol/L
Potassium	3.1 mmol/L
Chloride	105 mmol/L
Bicarbonate	27 mmol/L
Blood urea nitrogen	11 mg/dL
Creatinine	0.52 mg/dL
Glucose	92 mg/dL
Calcium	8.3 mg/dL
Total protein	6.9 g/dL
Albumin	3.9 g/dL
Aspartate aminotransferase	29 units/L
Alanine aminotransferase	23 units/L
Alkaline phosphatase	70 units/L
Total bilirubin	0.7 mg/dL

**Table 3:** Expanded testing results

Arsenic	6 ug/L
Lead	Not detected
Mercury	Not detected
Urine pregnancy	Negative
Troponin	<0.02 ng/mL
Human immunodeficiency virus assay	Not-reactive
Total serum triiodothyronine	151 ng/dL

**Table 4:** New York Heart Association functional classification of heart failure.

Class	Patient symptoms
I	No limitation of physical activity. Ordinary physical activity does not cause undue fatigue, palpitation, dyspnea (shortness of breath).
II	Slight limitation of physical activity. Comfortable at rest. Ordinary physical activity results in fatigue, palpitation, dyspnea (shortness of breath).
III	Marked limitation of physical activity. Comfortable at rest. Less than ordinary activity causes fatigue, palpitation, or dyspnea.
IV	Unable to carry on any physical activity without discomfort. Symptoms of heart failure at rest. If any physical activity is undertaken, discomfort increases.
	Objective assessment
	
A	No objective evidence of cardiovascular disease. No symptoms and no limitation in ordinary physical activity.
B	Objective evidence of minimal cardiovascular disease. Mild symptoms and slight limitation during ordinary activity. Comfortable at rest.
C	Objective evidence of moderately severe cardiovascular disease. Marked limitation in activity due to symptoms, even during less-than-ordinary activity. Comfortable only at rest.
D	Objective evidence of severe cardiovascular disease. Severe limitations. Experiences symptoms even while at rest.
